# Precision neuromodulation in epilepsy: from shared targets to personalized circuits

**DOI:** 10.1093/braincomms/fcaf327

**Published:** 2025-09-10

**Authors:** Sandipan Pati

**Affiliations:** Department of Neurology, University of Minnesota Comprehensive Epilepsy Program, University of Minnesota, MINCEP, Minneapolis, MN 55455, USA

## Abstract

This scientific commentary refers to ‘Thalamocortical network neuromodulation for epilepsy’, by Agashe *et al*. (https://doi.org/10.1093/braincomms/fcaf270).

**This scientific commentary refers to ‘Thalamocortical network neuromodulation for epilepsy’, by Agashe *et al*. (** https://doi.org/10.1093/braincomms/fcaf270 **).**

Despite remarkable advances in neuromodulation for neurological and psychiatric diseases, epilepsy remains one of the most compelling use cases for targeted electrical brain stimulation. Drug-resistant epilepsy affects nearly one-third of all individuals with epilepsy—amounting to over 15 million people globally—and for this population, electrical modulation of brain circuits offers a viable path when pharmacological and surgical options fail.^1^ However, a critical challenge looms: seizure semiology and their network substrates are diverse. Epilepsy is a syndrome, not a singular disease—its etiologies span cortical malformations, genetic channelopathies, acquired injuries, and immunologic disorders, all converging on the common symptom of seizures.^2^ Given this diversity, the notion that a single stimulation target or fixed protocol can serve all patients is no longer tenable.

Personalized stimulation is not a luxury—it is a necessity. Epileptogenic networks vary not only by etiology but also by seizure onset zone, propagation pattern and state dependence (e.g. sleep, wake).^3,4^ Mounting evidence suggests that neuromodulation is more effective when tailored to the patient’s individual seizure network rather than applied to population-based targets.^5-7^ For example, in patients whose seizure onset zones involve eloquent cortex, direct stimulation at or near the seizure focus (e.g. motor cortex, insula) may yield superior outcomes compared with remote thalamic stimulation.^8,9^ Similarly, in patients with multifocal or poorly localizable epilepsy, relying on a one-size-fits-all target like the anterior nucleus of the thalamus often results in partial or suboptimal control.^9^ Thus, identifying the right stimulation target—and equally, the right timing, frequency, and configuration—requires precision beyond current standard protocols.

So how do we move from conventional ‘shared-target’ stimulation to a truly personalized approach? One emerging strategy is to combine invasive network mapping with test stimulation during intracranial monitoring, allowing direct observation of target engagement, seizure suppression, and symptom modulation.^10-12^ The logic is simple: if a given node in the epileptic network can be perturbed acutely to modulate ictal activity or symptom severity, it may also serve as a durable site for chronic neuromodulation. This represents a paradigm shift—from selecting targets based solely on prior population studies, to dynamically testing network responsiveness in individual patients. Yet, despite its conceptual appeal, clinical implementation of this approach remains limited by several practical challenges—including the absence of standardized workflows, difficulty interpreting electrographic responses due to stimulation artifacts, safety and ethical concerns surrounding the sampling of deep structures like the thalamus, and a lack of robust long-term outcome data.

In this context, the study by Agashe *et al.*^13^ published in *Brain Communications* represents a notable advancement.^13^ The authors report a retrospective cohort of seven patients with multifocal or poorly localized epilepsy who underwent thalamocortical network neuromodulation using a novel 4-lead open-loop system. By implanting electrodes in both cortical and thalamic nodes identified through prior intracranial EEG (iEEG) monitoring and acute trial stimulation, the team delivered long-term stimulation targeting the patient’s specific seizure network. After a median follow-up of 17 months, all patients were classified as responders (≥50% reduction in seizure frequency), with a median seizure reduction of 93%, and significant improvements in seizure severity, life satisfaction, and sleep quality. Importantly, the procedure was well-tolerated, with no perioperative complications, and side effects were rare and reversible with parameter adjustment. The use of trial stimulation during iEEG was a key feature, allowing refinement of electrode placement based on network response.

This study makes several key contributions to the evolving field of personalized neuromodulation. First, it provides a compelling proof-of-concept that multi-node stimulation, guided by intracranial testing, can produce substantial seizure reduction—even in patients with complex, multifocal networks. Second, it demonstrates the feasibility of simultaneous cortical and thalamic targeting, advancing a network-based framework beyond the traditional dichotomy of selecting either a cortical or thalamic node. Third, it underscores the value of individualized network engagement, moving beyond anatomical heuristics to patient-specific functional validation. Perhaps most importantly, the study outlines a replicable clinical workflow: by integrating invasive monitoring, trial stimulation, and strategic chronic implantation, it offers a pragmatic blueprint for translating precision neuromodulation into routine practice.

What does this mean for practicing epileptologists? While the concept is exciting, implementation remains non-trivial. Most centers lack standardized workflows for trial stimulation, and there is no consensus on how to systematically test various targets or stimulation parameters during iEEG. Moreover, target selection is often guided by empirical heuristics rather than principled models of network dynamics or control. Some targets may lie at the seizure onset zone, others at propagation choke-points, and still others at intrinsic neuromodulatory choke-points (e.g. basal ganglia, cerebellum, subthalamic nucleus).^14,15^ Understanding which node to stimulate—and how—requires not only anatomical and electrophysiological mapping, but also conceptual frameworks for network control, feedback timing, and neuromodulatory physiology.

Looking ahead, several pressing questions must be addressed to move the field forward. Can we develop principled algorithms or biomarker-informed strategies to identify optimal stimulation targets and parameters tailored to each patient? Can acute stimulation responses—captured during trial stimulation—serve as reliable predictors of long-term outcomes with chronic neuromodulation? What roles do sleep state, circadian phase, and seizure cycle timing play in determining stimulation effectiveness? And critically, can we scale this approach beyond elite centers to broader clinical practice? Overcoming current limitations—such as the inability to record cortical responses during stimulation and the restricted channel counts of existing FDA-approved devices—will be essential. Addressing these technological and translational barriers is key to bridging the gap between research innovation and widespread clinical benefit for patients.

While the current study demonstrates feasibility and impact in a small but well-characterized cohort,^13^ larger prospective studies are essential to validate the safety, efficacy, and generalizability of this approach. The development of multicenter registries, standardized stimulation protocols, and wearable-integrated biomarker platforms could accelerate this transition.

In summary, this study^13^ is an important step toward the future of precision neuromodulation in epilepsy. Rather than selecting from a menu of pre-defined targets, we must now consider each patient’s seizure network as a dynamic system—one that can be probed, perturbed, and ultimately personalized. The field is not yet fully ready for widespread adoption of this model, but this work lights the path forward. As neuromodulation enters its second decade in epilepsy, the next wave must focus not only on *where* we stimulate—but *how* we personalize.
